# Characterization of the complete mitochondrial genome of the scale worm, *Eunoe nodosa* (Phyllodocida; Polynoidae) from the Beaufort Sea

**DOI:** 10.1080/23802359.2021.1955768

**Published:** 2021-09-06

**Authors:** Bo-Mi Kim, Sang-Eun Nam, Somyeong Lee, Ji-Hoon Kihm, Tae-Yoon S. Park, Jae-Sung Rhee

**Affiliations:** a Research Unit of Cryogenic Novel Material, Korea Polar Research Institute, Incheon, South Korea; b Department of Marine Science, College of Natural Sciences, Incheon National University, Incheon, South Korea; c Division of Earth Sciences, Korea Polar Research Institute, Incheon, South Korea; d Polar Science, University of Science and Technology, Daejeon, South Korea; e Research Institute of Basic Sciences, Incheon National University, Incheon, South Korea

**Keywords:** Complete mitogenome, Phyllodocida, Polynoidae, *Eunoe nodosa*, scale worm

## Abstract

To increase the mitogenome data available for robust phylogeny, we sequenced the complete mitochondrial DNA of the scale worm *Eunoe nodosa* (Sars, 1861) in the family Polynoidae of the order Phyllodocida. The complete mitogenome has 15,366 bp and has 28.9% A, 13.2% C, 19.0% G, and 38.8% T. Using MITOS and tRNAscan-SE, we identified the 13 typical protein-coding genes (PCGs), 2 ribosomal RNA (rRNA) genes, 22 transfer RNA (tRNA) genes, and a non-coding region. Phylogenomic analysis based on 27 in-group taxa belonging to five families of the subclass Errantia show congruence with the published phylogenetic relationship within the Polynoidae, in which *E. nodosa* lies in the clade of shallow water species.

The Suborder Aphroditiformia includes ‘scale worms,’ which show unique morphology in annelids, characterized by having dorsal elytra (Struck et al. 2011; Gonzalez et al. 2018). They are mostly macrofaunal benthic predators inhabiting all marine environments from shallow intertidal to deep sea including harsh environments (e.g. hydrothermal vents, volcanic seamounts, methane seeps), with a wide geographical distribution, ranging from tropics to polar regions (Rouse and Fauchald 1995; Zhang et al. 2017). Phylogenetic relationships within the Aphroditiformia have remained elusive for a long time due to the lack of proper genomic data, the excessive number of deep sea species, and the presence of scaleless group, Pisionidae (Rouse and Pleijel 2001; Struck et al. 2005; ; Norlinder et al. 2012). Recently, however, genomic data has begun to elucidate the relationships within the Aphroditiformia (Gonzalez et al. 2018; Zhang et al. 2018; Hatch et al. 2020).

The scale worm *Eunoe nodosa* (M. Sars, 1861) is widely distributed in the Arctic, Northern Atlantic, Mediterranean, and Northern Pacific with depth range up to 1260 m (Drumm et al. 2016). There is no information on complete mitogenome in the genus *Eunoe*, and several incomplete PCGs (e.g. *COI*, *16S rRNA*, *18S rRNA*) of the *Eunoe* species have been registered at NCBI GenBank. In this study, a live specimen of *E. nodosa* was collected from the Beaufort Sea (69°52'N, 139°03'W) in 2017 using a remotely operated underwater vehicle (ROV) of the Monterey Bay Aquarium Research Institute (MBARI). Its distribution in the Beaufort Sea was previously described (Miller 2012). The sample is deposited in the Korea Polar Research Institute (Species ID: Annelid-04; Specimen ID: KOPRI-Benthos-4; https://antagen.kopri.re.kr; Dr. Bo-Mi Kim; bomikim@kopri.re.kr). Genomic DNA was isolated from the muscle tissue of a single individual using a DNeasy Blood and Tissue kit (Qiagen, Hilden, Germany) according to the manufacturer’s instructions. Next-generation sequencing was conducted to obtain a circular mitogenome using the protocols based on a previous study (Nam et al. 2021). TruSeq DNA Sample Preparation Kit (Illumina, San Diego, CA, USA) was used for sequencing using the Illumina HiSeq sequencer. The sequencing library was prepared by random fragmentation of the DNA sample, followed by 5′ and 3′ adapter ligation. Raw reads were obtained from the sample that passed the quality control check in the Illumina HiSeq platform (Illumina) at Macrogen, Inc. (Seoul, South Korea). Adapter sequences, low quality reads, reads with >10% of unknown bases, and ambiguous bases were removed to obtain high quality assembly. After the quality check process, a total of 14,484,343 filtered reads were obtained from 16,493,324 raw reads. Thereafter, *de novo* assembly was conducted with various k-mers using SPAdes (Bankevich et al. 2012), and a circular contig of the *E. nodosa* mitogenome was obtained. The resulting contig consensus sequence was annotated using MITOS2 (Bernt et al. 2013) and tRNAscan-SE 2.0 (Lowe and Eddy, 1997). Further, BLAST searches confirmed the identity of the genes (http://blast.ncbi.nlm.nih.gov).

The complete mitogenome of *E. nodosa* was 15,366 bp in length (GenBank accession no. MW557378) carrying the typical composition including 13 PCGs, 22 tRNAs, two rRNAs, and an intergenic region. The nucleotide composition was highly biased toward A + T nucleotides (67.7%), with the percentage of A, T, C, and G being 28.9, 38.8, 13.2, and 19.0%, respectively. The *COI* sequence showed the highest similarity (99.7%) to the *COI* sequence of *E. nodosa* that was previously registered at GenBank (656 bp; GenBank accession no. HQ024300; Carr et al. 2011). The overall genomic architecture of the *E. nodosa* mitochondrion was similar to the previously published mitogenomes of shallow-water polynoids (*Halosydna* sp., *Melaenis* sp., and *Lepidonotus* sp.), showing difference in gene order and orientation from those of deep sea polynoids, abranchiates (*Levensteiniella iris*, *Lepidonotopodium okinawae*) and branchiates (*Branchipolynoe* sp. and *Branchinotogluma japonicus*). This discrepancy would be attributable to the unique mitochondrial genome evolution of the deep sea species (Zhang et al. 2018).

We constructed the phylogenetic topology of 33 annelid species using the concatenated set of the whole 13 PCG sequences, with two sipunculan species as an outgroup (Figure 1). Overall, the sister-group relationship of Errantia and Sedentaria complies well with the previously established phylogeny (Weigert and Bleidorn 2016). In the phylogenetic tree made from the 26 published mitogenomes of Errantia, *E. nodosa* lies within the family Polynoidae, forming a cluster with *Melaenis* sp. As shown in the recent phylogenetic analysis (Zhang et al. 2018), deep sea polynoids (abranchiate and branchiate species) form a well-supported monophyletic clade, with the shallow-water species including *E. nodosa* forming the sister clade. As this study was not accompanied by morphological observations, incorporation of the morphological information would be needed for future taxonomic studies of the genus *Eunoe*.

**Figure 1.**
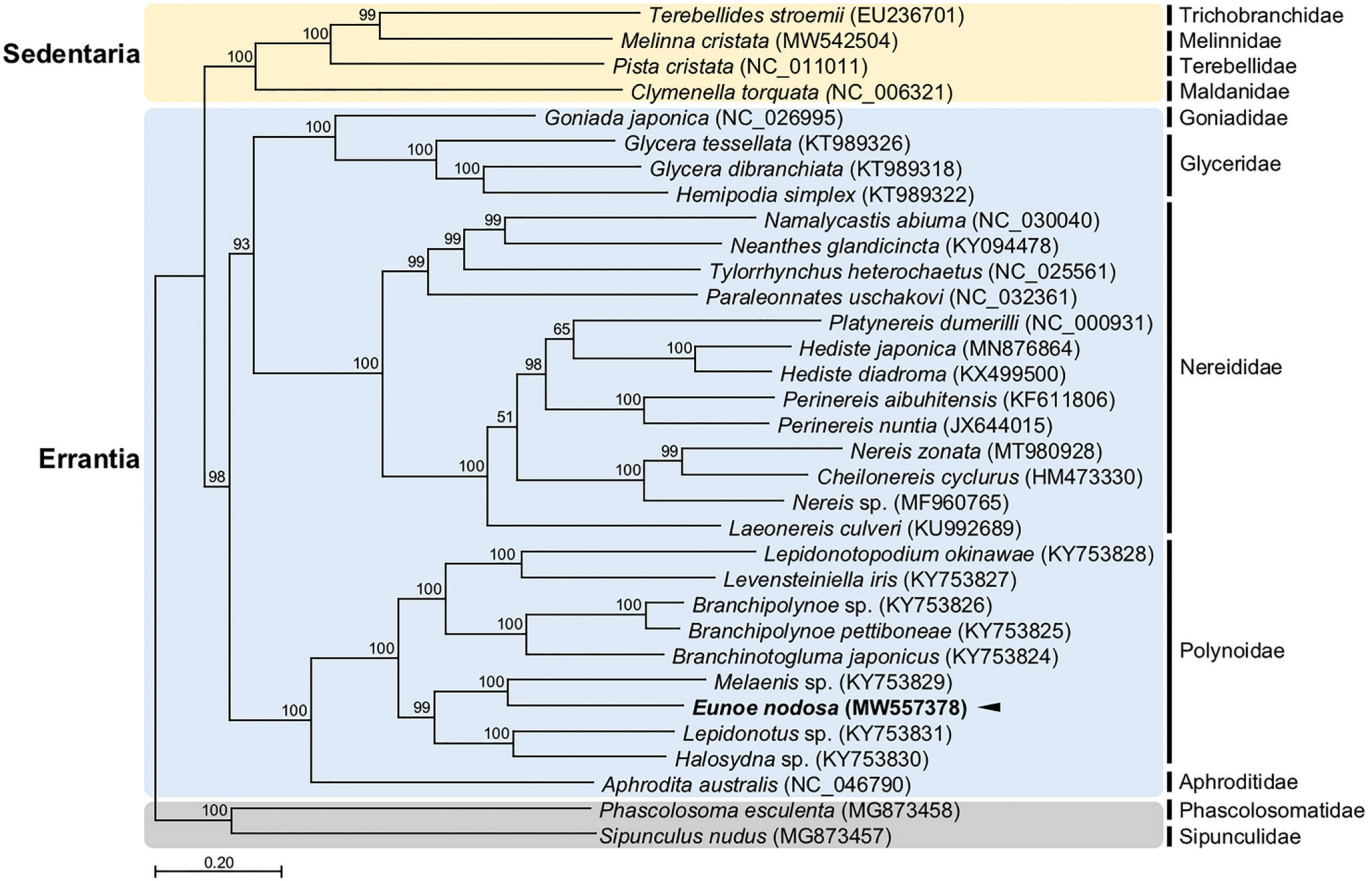
Maximum-likelihood (ML) phylogeny of 26 published mitogenomes from Errantia including *E. nodosa* and 4 registered mitogenomes of Sedentaria species, and two Sipuncula species as an outgroup based on the concatenated nucleotide sequences of protein-coding genes (PCGs). The phylogenetic analysis was performed using the maximum likelihood method, GTR + G + I model with a bootstrap of 1,000 replicates. Numbers on the branches indicate ML bootstrap percentages. DDBJ/EMBL/Genbank accession numbers for published sequences are incorporated. The black triangle means the scale worm analyzed in this study.

## Data Availability

BioProject, BioSample, and SRA accession numbers are https://www.ncbi.nlm.ni h.gov/bioproject/PRJNA699106, https://www.ncbi.nlm.nih.gov/biosample/SAMN17766237, and https://www.ncbi.nlm.nih.gov/sra/?term=SRR14996616, respectively. The data that support the findings of this study are openly available in the National Center for Biotechnology Information (NCBI) at https://www.ncbi.nlm.nih.gov, with an accession number MW557378.

## References

[CIT0001] Bankevich A, Nurk S, Antipov D, Gurevich AA, Dvorkin M, Kulikov AS, Lesin VM, Nikolenko SI, Pham S, Prjibelski AD, Pyshkin AV, et al. 2012. SPAdes: a new genome assembly algorithm and its applications to single-cell sequencing. J Comput Biol. 19(5):455–477. 22506599 10.1089/cmb.2012.0021 PMC3342519

[CIT0002] Bernt A, Donath A, Jühling F, Externbrink F, Florentz C, Fritzsch G, Pütz J, Middendorf M, Stadler PF. 2013. MITOS: improved de novo metazoan mitochondrial genome annotation. Mol Phylogenet Evol. 69(2):313–319. 22982435 10.1016/j.ympev.2012.08.023

[CIT0003] Carr CM, Hardy SM, Brown TM, Macdonald TA, Hebert PD. 2011. A tri-oceanic perspective: DNA barcoding reveals geographic structure and cryptic diversity in Canadian polychaetes. PLOS One. 6(7):e22232. 21829451 10.1371/journal.pone.0022232 PMC3136506

[CIT0004] Drumm DT, Maslenikov KP, Van Syoc R, Orr JW, Lauth RR, Stevenson DE, Pietsch TW. 2016. An annotated checklist of the marine macroinvertebrates of Alaska. NOAA Prof Paper NMFS. 19:289.

[CIT0005] Gonzalez BC, Martínez A, Borda E, Iliffe TM, Eibye-Jacobsen D, Worsaae K. 2018. Phylogeny and systematics of Aphroditiformia. Cladistics. 34(3):225–259. 10.1111/cla.12202 34645076

[CIT0006] Hatch AS, Liew H, Hourdez S, Rouse GW. 2020. Hungry scale worms: phylogenetics of Peinaleopolynoe (Polynoidae, Annelida), with four new species. Zookeys. 932:27–74. 32476973 10.3897/zookeys.932.48532 PMC7237507

[CIT0007] Lowe TM, Eddy SR. 1997. tRNAscan-SE: a program for improved detection of transfer RNA genes in genomic sequence. Nucleic Acids Res. 25(5):955–964. 9023104 10.1093/nar/25.5.955 PMC146525

[CIT0008] Miller R. 2012. The museum collection database, fisheries and oceans Canada digital collections. Quebec: Maurice Lamontagne Institute.

[CIT0009] Nam S-E, Lee S, Park T-YS, Rhee J-S. 2021. Complete mitochondrial genome of the marine polychaete, *Nereis zonata* (Phyllodocida, Nereididae) isolated from the Beaufort Sea. Mitochondr DNA B Resour. 6(1):231–233. 10.1080/23802359.2020.1861994 PMC7832487 33537452

[CIT0010] Norlinder E, Nygren A, Wiklund H, Pleijel F. 2012. Phylogeny of scale-worms (Aphroditiformia, Annelida), assessed from 18SrRNA, 28SrRNA, 16SrRNA, mitochondrial cytochrome c oxidase subunit I (COI), and morphology. Mol Phylogenet Evol. 65(2):490–500. 22789762 10.1016/j.ympev.2012.07.002

[CIT0011] Rouse GW, Fauchald K. 1995. The articulation of annelids. Zool Scripta. 24(4):269–301.

[CIT0012] Rouse GW, Pleijel F. 2001. Polychaetes. New York: Oxford University Press.

[CIT0013] Struck TH, Paul C, Hill N, Hartmann S, Hösel C, Kube M, Lieb B, Meyer A, Tiedemann R, Purschke G, et al. 2011. Phylogenomic analyses unravel annelid evolution. Nature. 471(7336):95–98. 21368831 10.1038/nature09864

[CIT0014] Struck TH, Purschke G, Halanych KM. 2005. A scales scale worm: molecular evidence for the phylogenetic placement of *Pisione remota* (Pisionidae, Annelida). Mar Biol Res. 1(4):243–253.

[CIT0015] Weigert A, Bleidorn C. 2016. Current status of annelid phylogeny. Org Divers Evol. 16(2):345–362.

[CIT0016] Zhang Y, Sun J, Chen C, Watanabe HK, Feng D, Zhang Y, Chiu JMY, Qian P-Y, Qiu J-W. 2017. Adaptation and evolution of deep-sea scale worms (Annelida: Polynoidae): insights from transcriptome comparison with a shallow-water species. Sci Rep. 7:46205. 28397791 10.1038/srep46205 PMC5387418

[CIT0017] Zhang Y, Sun J, Rouse GW, Wiklund H, Pleijel F, Watanabe HK, Chen C, Qian P-Y, Qiu J-W. 2018. Phylogeny, evolution and mitochondrial gene order rearrangement in scale worms (Aphroditiformia, Annelida). Mol Phylogenet Evol. 125:220–231. 29625228 10.1016/j.ympev.2018.04.002

